# The Economic Burden of Hospital Costs on Families With Type 1 Diabetes Mellitus Children: The Role of Medical Insurance in Shandong Province, China

**DOI:** 10.3389/fpubh.2022.853306

**Published:** 2022-05-06

**Authors:** Siyuan Wang, Yawei Guo, Elizabeth Maitland, Stephen Nicholas, Jingjie Sun, Anli Leng

**Affiliations:** ^1^Faculty of Business and Economics, University of Melbourne, Melbourne, VIC, Australia; ^2^Taiyuan Municipal Health Commission, Taiyuan, China; ^3^School of Management, University of Liverpool, Liverpool, United Kingdom; ^4^Australian National Institute of Management and Commerce, Sydney, NSW, Australia; ^5^Newcastle Business School, University of Newcastle, Newcastle, NSW, Australia; ^6^Shandong Health Commission Medical Management Service Center, Jinan, China; ^7^School of Political Science and Public Administration, Shandong University, Qingdao, China; ^8^Center for Health Preferences Research, Shandong University, Jinan, China

**Keywords:** hospitalization costs, childhood type 1 diabetes, T1DM children, economic burden, medical insurance

## Abstract

**Objective:**

This study estimates the economic burden imposed on families by comparing the hospitalization costs of T1DM children with and without medical insurance in Shandong province.

**Methods:**

Our data comprised 1,348 T1DM inpatient records of patients aged 18 years or younger from the hospitalization information system of 297 general hospitals in 6 urban districts of Shandong Province. Descriptive statistics are presented and regression analyses were conducted to explore the factors associated with hospitalization costs.

**Results:**

Children with medical insurance had on average total hospitalization expenditures of RMB5,833.48 (US$824.02) and a hospitalization stay of 7.49 days, compared with the children without medical insurance who had lower hospitalization expenditures of RMB4,021.45 (US$568.06) and an average stay of 6.05 days. Out-of-pocket expenses for insured children were RMB3,036.22 (US$428.89), which is significantly lower than that of the uninsured children (*P* < 0.01). Out-of-pocket (OOP) expenditures accounted for 6% of the annual household income of insured middle-income families, but rose to a significant 25% of the annual income for low-income families. These OOP expenditures imposed a heavy economic burden on families, with some families experiencing long-term financial distress. Both insured and uninsured families, especially low-income families, could be tipped into poverty by hospitalization costs.

**Conclusion:**

Hospitalization costs imposed a significant economic burden on families with children with T1DM, especially low-income insured and uninsured families. The significantly higher hospitalization expenses of insured T1DM children, such as longer hospitalization stays, more expensive treatments and more drugs, may reflect both excess treatment demands by parents and over-servicing by hospitals; lower OOP expenses for uninsured children may reflect uninsured children from low-income families forgoing appropriate medical treatment. Hospital insurance reform is recommended.

## Introduction

Diabetes mellitus is a complex chronic non-communicable disease with acute and long-term consequences and it is becoming an increasingly important global health issue (1, 2). The global prevalence of diabetes is expected to increase from 171 million in 2000–366 million by 2030 (3, 4). In 2015, the absolute global cost of diabetes was US$1.31 trillion or 1.8% of global gross domestic product, and is projected to reach US$2.5 trillion by 2030 based on past trends (5, 6). Moreover, the health complications arising from diabetes, such as cardiovascular disease, pose an obvious and rising health cost on diabetes sufferers and a nation's health care system (7).

A unique subset of diabetes sufferers are children. The changes in children's lifestyle and entertainment trends, high calorie intake and reduction in energy expenditure has contributed to an obesity pandemic and increased prevalence of type 1 diabetes (T1DM) in children (8, 9). A study in The Lancet showed that the incidence of T1DM continues to increase, especially in young children from high- and middle-income countries (10). According to a study comprising 13 cities from different Chinese provinces, the estimated incidence of T1DM for ages 0–14 years old in Jinan, Shandong's provincial capital, was 2.18 per 100,000 person years (11). The same study estimated T1DM incidence rates of 1.02 per 100,000 person years for 15–29 year olds, and 0.51 per 100,000 person years for those older than 30 years old between 2010 and 2013 (11). The evidence shows that the incidence of childhood type 1 diabetes mellitus in those <18 years old is significantly higher than other age groups in China. This trend is not limited to China. An America study argued that over the last 3 decades, T1DM, a disease which previously only affected adults, had an increasingly noticeable prevalence among children and adolescents (12). Evidence from Africa showed that the prevalence of T1DM in children under 5 was increasing (13) and in Taiwan between 1999 and 2010, the incidence rate of type 1 diabetes mellitus was highest when the age was <15 years old (14). The growing number of children with diabetes pose a global health economic challenge, including financial burden to a country's health system and economic stress to families with T1DM children.

Shandong was one of China's developed eastern coast industrial provinces, where roughly 40% of the adult population had 12 years or more of education and the province's per capita gross domestic product of RMB72,200.00 (US$11,352.00) ranked the 11th highest in China (15). According to China's seventh national census for Shandong Province, the number of children between 0 and 14 years old was 19.01 million, roughly 18.8% of the total population, comprising 64.01 million urban residents and 37.51 million rural residents. Due to the specific needs of a child's growth and development, the treatment for T1DM children is quite different from that of adults, and the cost of medical treatment is relatively high, which imposes a heavy economic burden on families, the health system and the medical insurance system (16). China has not yet established a comprehensive government-funded child health insurance system that could meet the medical welfare needs of this growing group of T1DM children and protect their families from the economic burden of T1DM hospital costs. We explore the hospitalization costs incurred by T1DM children and the economic burden on families, with and without medical insurance.

Early diabetes studies focused on the composition of hospitalization costs for adult diabetic patients (17), explored the factors which influenced these hospitalization costs (18, 19) and quantified the economic burden of hospitalization cost for adult patients (20–23). One study distinguished the hospitalization costs between adult sufferers with and without medical insurance (24). In China, there is a gap in the literature on the hospitalization costs imposed on families with children with type 1 diabetes mellitus. This lack of data constrains policymakers in their development of nationwide child diabetes strategies and proposed changes to China's national health insurance schemes (25). Using data from Shandong province, we address this lacuna by conducting a cross-section study comparing the hospitalization costs burden on families with T1DM children, with and without medical insurance.

## Materials and Methods

### Settings and Data Source

**Figure 1:**
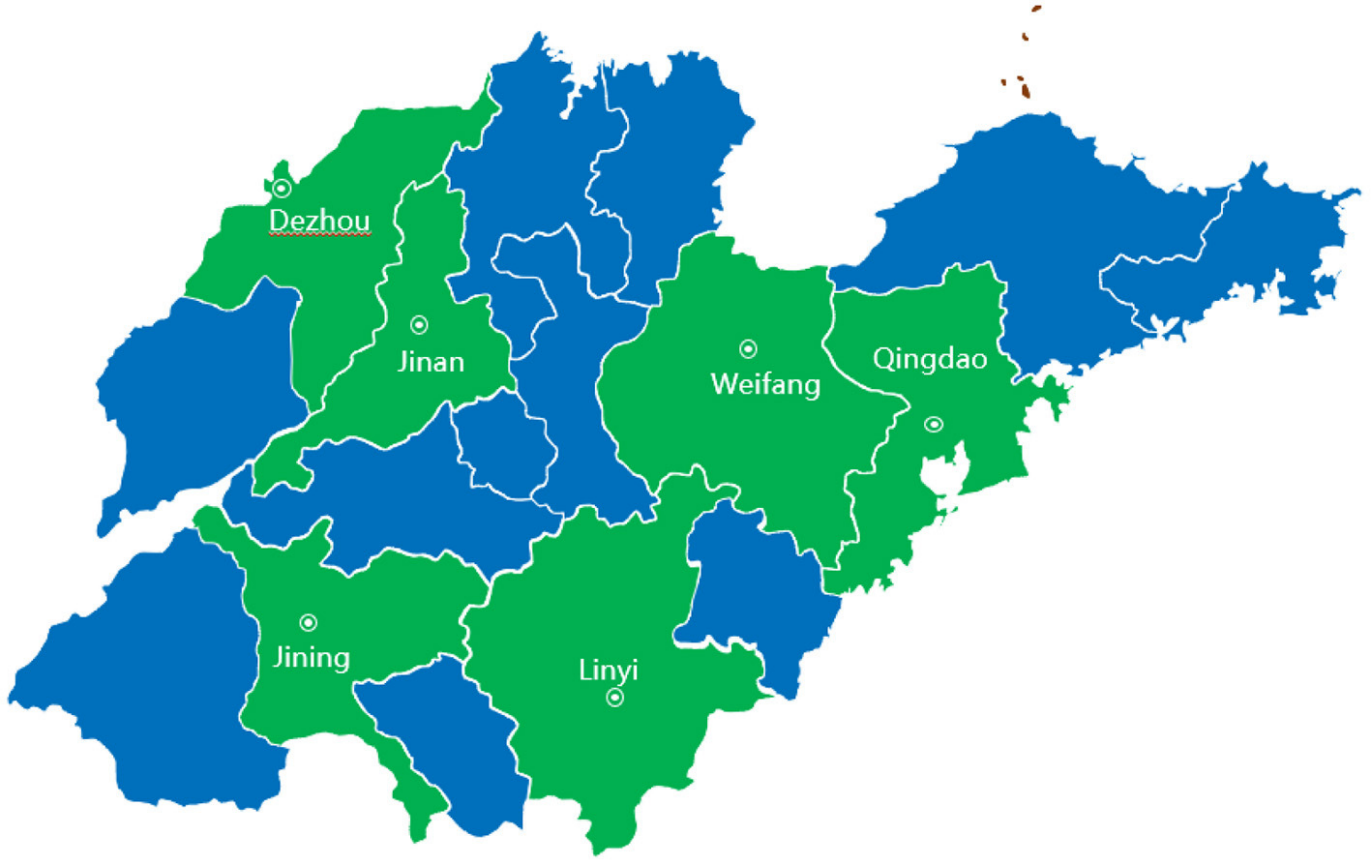
Sampling cities of this study in Shandong Province.

In Shandong Province, a 3-stage cluster sampling was used to select sampling sites. First, all cities in Shandong Province were stratified into three groups by the status of their economic development. As shown in Figure 1, two cities were selected in each group, with Qingdao and Weifang being selected from the eastern region, Jinan and Linyi being selected from the middle region and Dezhou and Jining being selected from the western region. We then selected four districts from each city on the basis of GDP per capita. Finally, six sub-districts or townships were selected from each chosen district. This yielded 6 cities, 24 districts and 192 sub-districts or townships as sampling sites. From these sampling sites, we chose 297 medical facilities as sampling institutions, including general hospitals, traditional Chinese medicine hospitals, maternal and child health hospitals, specialist hospitals, community health service centers and township health centers. From 297 medical institutions, we extracted data on all inpatients from January 1 to December 31, 2017.

### Study Population

Hospitalized T1DM inpatients were identified when the primary discharge diagnosis corresponded to the 10th edition of the International Classification of Diseases (ICD-10) E10 diabetes codes. Using the electronic system of the medical institution, trained medical staff uniformly reported ICD-10 codes. In order to reduce any errors, all ICD-10 codes for 2017 were proofread using a special computer program developed by the National Health and Wellness Development Committee. Inpatients aged <18 years with at least 1 day of hospitalization were included, yielding a sample of 1,348 qualified type 1 diabetes mellitus children in 297 medical institutions across Shandong province. All inpatients had complete information recorded regarding their demographics, regional distribution of cities, comorbidities, sex, age, health insurance, hospitalization costs, insurance reimbursements and the information about the health facilities.

China is in the process of integrating the New Cooperative Medical Scheme (NCMS) for its rural population, and the Urban Residents Basic Medical Insurance System (URBMI) for its urban out-of-workforce population. This involves integrating urban children, students, the unemployed and the disabled into the Urban and Rural Medical Insurance Scheme (IURMI). By the end of 2020, the National Healthcare Security Administration reported 1.02 billion IURMI participants, of which 246 million (24.2%) were children (26). Compared to adults under the IURMI, children experienced lower participation rates, lesser compensation for medical expenses and differentiated levels in medical security protection caused by varying insurance policies (27). In addition, child T1DM inpatients were likely to have lower hospital expenses compared to adults, but will incur significant on-going costs during their lifetime (27). Shandong province completed China's IURMI reforms in 2015, with children under the age of 18 only having IURMI or no insurance. Compared to insured children, uninsured children came from households with lower self-reported family income, higher non-enrollment in education, migrants and poor parental awareness and decision-making (28, 29).

### Statistical Analysis

Statistical analysis was performed using STATA Version 14.0 and statistical significance was set at the 5% significance level. For continuous variables, the *p*-value was calculated using Student's *t*-test; and for categorical variables, the *p*-value was calculated using the chi-square test. We used a multivariate linear regression model to analyze relationships between hospitalization costs and potential influential factors. We specified the following regression model:


$$\begin{array}{l} \text{Y}={\text{β}}_{0}+{\text{β}}_{1}{\text{X}}_{1}+{\text{β}}_{2}{\text{X}}_{2}+\cdot \cdot \cdot +{\text{β}}_{\text{m}}{\text{X}}_{\text{m}}+\text{e} \end{array}$$

where the dependent variable (Y) was hospitalization costs and the independent variables were age, sex, with or without medical insurance, severity of illness represented by with or without comorbidities, regional distribution of cities, geographical location of the medical institution and whether the medical institution was public or private.

## Results

### Basic Information of the Participants

As displayed in Table 1, the study included a total of 1,348 inpatient children with type 1 diabetes, with a mean age of 8.97 years ± 0.16 and 52.89% were male. Of these child inpatients, 998 or 74.04%, were identified as having medical insurance, with the remaining 350 (25.96%) without medical insurance. We categorized children into three age groups, where those <6 years (34.87%), between 6 and 11 years (31.90%) and 12 years and older (33.23%), each accounting for about one third of the sample. Children under 6 most likely to be uninsured. The proportion of child inpatients from urban areas (87.98%) was significantly larger than rural inpatients (12.02). In our sample, two-thirds (65.28%) of the inpatient children were without comorbidities; 1,319 were treated at national public hospitals and 29 were treated at private health facilities; and the number of patients from the eastern, middle and western region was roughly the same.

**Table 1:** Baseline and demographic characteristics.

		**Medical insurance**			
	***N* (%)**	**No (%)**	**Yes (%)**	***X* ^2^**	***P***
**Observations**	1,348 (100)	350 (25.96)	998 (74.04)		
**Sex**				0.3679	0.5440
Males	713 (52.89)	190(26.65)	523 (73.35)		
Females	635 (47.11)	160(25.20)	475 (74.80)		
**Age**				25.7463	**0.0000**
0–5	470 (34.87)	152 (32.34)	318 (67.66)		
6–11	430 (31.90)	118 (27.44)	312 (72.56)		
12–18	448 (33.23)	80 (17.86)	368 (82.14)		
**Location**				114.2038	**0.0000**
Urban	1186 (87.98)	252 (21.25)	934 (78.75)		
Rural	162 (12.02)	98 (60.49)	64 (39.51)		
**Comorbidity**				14.8313	**0.0000**
Yes	468 (34.72)	92 (19.66)	376 (80.34)		
No	880 (65.28)	258 (29.32)	622 (70.68)		
**Regional distribution**				7.3364	**0.0260**
East	460 (34.12)	140 (30.43)	320 (69.57)		
Middle	538 (39.91)	129 (23.98)	409 (76.02)		
West	350 (25.97)	81 (23.14)	269 (76.86)		
**Type of health facility**				0.0406	0.8400
Public	1,319 (97.85)	342 (25.93)	977 (74.07)		
Private	29 (2.15)	8 (27.59)	21 (72.41)		

*Bold values are statistically significant p values at the 5% level*.

### Hospitalization Costs of Type 1 Diabetes in Children With and Without Medical Insurance

As shown in Table 2, the type 1 diabetes mellitus inpatients with medical insurance had significantly higher total hospital expenditure (RMB5,833.48 ± 220.63/US$824.8 ± 31.2) than those without medical insurance (RMB4,021.45 ± 176.66/US$568.6 ± 24.98, *p* < 0.001) and had a longer stay in hospital (7.49 ± 0.17 days) than those without medical insurance (6.05 ± 0.24 days, *p* < 0.001). Breaking down the specific hospital expenditures, the treatment costs, drug costs, bed fees, diagnosis fees, check fees and test expenditure were all significantly higher for children with medical insurance than those without medical insurance. Other health cost, including surgery costs and nursing costs were not significantly different between the insured and uninsured children.

**Table 2:** Hospitalization costs of children with type 1 diabetes (RMB).

**Variable**	**All (mean ±SD)**	**Inpatient with medical insurance (mean ±SD)**	**Inpatient without medical insurance (mean ±SD)**	***t*-value**	***p*-value**
Age	8.97 ± 0.16	9.39 ± 0.18	7.79 ± 0.29	4.4983	**0.0000**
Length of stay	7.11 ± 0.14	7.49 ± 0.17	6.05 ± 0.24	4.5645	**0.0000**
Total costs	5,363.00 ± 171.00	5,833.48 ± 220.63	4,021.45 ± 176.66	4.6819	**0.0000**
Treatment costs	824.87 ± 40.60	908.89 ± 52.11	585.30 ± 46.41	3.5095	**0.0005**
Drug costs	934.80 ± 42.20	1,041.16 ± 55.29	631.48 ± 34.80	4.2842	**0.0000**
Bed fees	325.71 ± 11.37	340.25 ± 13.59	284.28 ± 20.25	2.1614	**0.0308**
Diagnosis fees	122.31 ± 5.23	134.33 ± 6.35	88.05 ± 8.51	3.9034	**0.0001**
Check-up fees	373.43 ± 18.02	397.85 ± 22.76	303.79 ± 24.23	2.2925	**0.0220**
Surgery costs	75.64 ± 11.30	84.23 ± 14.66	51.13 ± 12.07	1.2843	0.1993
Test costs	1,064.74 ± 33.97	1,154.19 ± 42.29	809.67 ± 48.32	4.4776	**0.0000**
Nursing fees	163.85 ± 6.92	166.48 ± 8.54	156.35 ± 10.86	0.6416	0.5213
Other costs	541.51 ± 46.16	563.61 ± 60.59	478.47 ± 41.93	0.8086	0.4189
Out-of-pocket costs	3,292.03 ± 89.26	3,036.22 ± 102.25	4,021.45 ± 176.66	4.8803	**0.0000**

*Bold values are statistically significant p values at the 5% level*.

### Factors Associated With Hospitalization Costs

Table 3 shows the results of the multivariate regression analysis for the major factors associated with hospitalization costs, listed in the decreasing order of their absolute *t*-value. The most significant influential factors for hospitalization costs were with or without medical insurance, urban-rural location of the medical institution and severity of illness represented by with or without comorbidities. Sex, regional distribution of cities, age group and type of medical institution were not significantly related to the total cost of hospitalization.

**Table 3:** Regression coefficients and standard errors for major factors associated with hospitalization costs.

**Factors**	**Coefficients**	**SE**	***t*-value**	***p*-value**
Comorbidities	3,078.3660	353.5739	8.7100	**0.0000**
Location	2,764.0280	535.8362	5.1600	**0.0000**
Medical insurance	949.8624	394.1617	2.4100	**0.0160**
Regional distribution	241.6070	218.1295	1.1100	0.2680
Age-group	213.2196	204.5707	1.0400	0.2970
Gender	140.4322	327.9564	0.4300	0.6690
Type of health facility	247.9743	1,137.1560	0.2200	0.8270

*Bold values are statistically significant p values at the 5% level*.

## Discussion

This is the first cross-sectional study of the hospital costs incurred by Chinese type 1 diabetes mellitus children with or without medical insurance. The results show that the median hospitalization costs for T1DM children was RMB5,363.00 (US$757.79), which was lower than RMB7,996.11 (US$1,162.53) found in people of all ages with diabetes in Beijing (17). Median hospitalization costs was also lower than the US$863.2 found by a rural southwest China study for population aged 18 or over (4) and lower than the US$1,655 found for adults in a provincial capital city in east China (30). A Chinese study estimated the median hospitalization costs for diabetes mellitus adult inpatients with medical insurance was RMB9,458 (US$1,407) and RMB9,104 (US$1,354) without medical insurance (24). The international average annual treatment and management cost for people with diabetes was US$1,622–2,886 per person in 2015 (31). Our focus on children with type 1 diabetes had its own specific characteristics: compared with adults, children with diabetes have fewer complications, lower severity and shorter hospital stays and, therefore, lower hospitalization costs. Of course, we did not calculate the ongoing costs of child diabetes sufferers, where under 18 years old children would accumulate hospitalization expenses over their lifetime.

Children with medical insurance had significantly higher total hospital expenditures for various services, such as treatment, drug costs, bed fees, diagnosis fees, check-up fees and test costs, and longer hospital stays than children without insurance. Part of this difference was related to parents with medical insurance seeking better quality and a greater quantity of medical services and treatments for their children (24). From the supply side, children with insurance may be over-serviced, reflected in excessive hospital services and longer hospital stays. Parental demands of insured children for more medical care, and more frequent doctor visits, will encourage over-servicing, including more treatments, drugs, surgery and test fees. For families without medical insurance, we suggest a medical services self-selection bias effect, with parents demanding lower levels of hospital care and shorter stays for uninsured children.

From Table 2, diabetes mellitus inpatients with medical insurance had significantly lower out-of-pocket expenses (RMB3,036.22 ± 102.25) than inpatients without medical insurance (RMB4,021.45 ± 176.66, *p* < 0.001), which is consistent with previous studies (24, 32). Lower out-of-pocket expenses also encouraged higher consumption of hospital stays and medical treatments for insured inpatients. It was also the case that children with poor health were more likely to participate in medical insurance schemes (33), which makes some medically insured children more likely to incur excessive hospitalization costs due to greater severity of illness caused by more comorbidities. Of course, not all costs were related to insurance status, with surgery, nursing and other costs not significantly different for insured and uninsured inpatients.

Medical insurance reduced, but did not do away with, the economic burden of hospital costs on families, while those without medical insurance faced significant hospital and health care costs that severely strained average household budgets. In 2015, the average annual income of middle-income households in China was RMB49,809.73 (US$7,239.05) and that of low-income households was only RMB12,048.79 (US$1,751.10). Out-of-pocket expenses of RMB3,036.22 (US$429.01) for insured middle-income families accounted for about 6% of yearly family income, but 25% of low-income insured families' yearly family income. For uninsured families, out-of-pocket expenses of RMB4,021.45 (US$568.23) for middle-income families was 8% of the yearly family income and 33% of the yearly family income for low-income families. Both insured and uninsured families with type 1 diabetes children faced an economic burden imposed by hospital costs, with low-income families' financial resources severely impacted by out-of-pocket hospital expenses. Many insured and uninsured families would deplete family savings, sell assets or borrow to pay for out-of-pocket hospitalization expenses. Some families faced long-term economic distress, and some families were tipped into poverty, when over 40% of their yearly non-food budget was accounted for by out-of-pocket hospital expenses (34).

There are some limitations in our study. First, it was a cross-section study, and the relationship between hospitalization costs and other factors cannot be interpreted as cause and effect. Second, there was a lack of information on the detailed symptoms of inpatients, so we were unable to analyze whether the hospital costs were “reasonable” for the treatment required. Additional data on the inpatients' medical conditions should be collected in follow-up studies. Our data only included single visit hospitalization costs. Annual hospitalization costs of insured and non-insured inpatients should be collected in future studies. Also, the addition of hospital levels in the analysis of cost factors should be included in future studies. Further studies should also include more socioeconomic factors, including family income and household savings data, which will allow the full economic impact of the hospitalization costs of T1DM children on families to be evaluated, and also allow more in-depth policy research and health economics evaluations. While we are confident that we drew a representative sample from Shandong province, data from other provinces, especially in less developed regions, are required to confirm our data are representative of all Chinese provinces.

## Conclusions

This study assessed the economic burden imposed on families by comparing the hospitalization costs of T1DM children with and without medical insurance in Shandong province. Based on our results, we recommend further reform to the medical insurance system in China, providing equity and better accessibility to more families; providing additional protection to vulnerable low-income families; and to allow families to purchase child medical insurance. The different cost structures between insured and uninsured type 1 diabetes mellitus children suggest the need for further investigations of treatment regimes, including over-demand by parents for treatment of their children and over-supply of treatment by medical staff. Most worrying is the self-treatment bias for children by uninsured parents. Many parents with uninsured children faced high out-of-pocket expenses, which may have encouraged parents to curtail the medical treatment of their children. This is supported by the evidence that uninsured children had shorter hospital stays, less drugs and fewer tests, which may have imposed a significant potential health risk to T1DM children. Inadequate childhood diabetes treatment likely increased the costs of adulthood diabetes treatment, with negative effects on both individual diabetes sufferers and on the Chinese health system.

## Data Availability Statement

The raw data supporting the conclusions of this article will be made available by the authors, without undue reservation.

## Author Contributions

SW and YG contributed to the conception and design of the manuscript. YG wrote the first draft. SW wrote sections of the manuscript. JS contributed toward the article by collecting data. SN, EM, and AL contributed to the manuscript revision. All authors read and approved the final version of the manuscript.

## Funding

This work was supported by the National Natural Science Foundation of China (Grant Number 72004117) and China Postdoctoral Science Foundation (Grant Number 2019M662392).

## Conflict of Interest

The authors declare that the research was conducted in the absence of any commercial or financial relationships that could be construed as a potential conflict of interest.

## Publisher's Note

All claims expressed in this article are solely those of the authors and do not necessarily represent those of their affiliated organizations, or those of the publisher, the editors and the reviewers. Any product that may be evaluated in this article, or claim that may be made by its manufacturer, is not guaranteed or endorsed by the publisher.
